# Sudden Event Recognition: A Survey

**DOI:** 10.3390/s130809966

**Published:** 2013-08-05

**Authors:** Nor Surayahani Suriani, Aini Hussain, Mohd Asyraf Zulkifley

**Affiliations:** Smart Engineering System Research Group, Department of Electrical, Electronic and Systems Engineering, Faculty of Engineering and Built Environment, Universiti Kebangsaan Malaysia, Bangi 43600, Malaysia; E-Mails: nsuraya@siswa.ukm.edu.my (N.S.S.); asyraf@eng.ukm.my (M.A.Z.)

**Keywords:** sudden event recognition, motion pattern, video surveillance, foreground detection, object tracking, object recognition

## Abstract

Event recognition is one of the most active research areas in video surveillance fields. Advancement in event recognition systems mainly aims to provide convenience, safety and an efficient lifestyle for humanity. A precise, accurate and robust approach is necessary to enable event recognition systems to respond to sudden changes in various uncontrolled environments, such as the case of an emergency, physical threat and a fire or bomb alert. The performance of sudden event recognition systems depends heavily on the accuracy of low level processing, like detection, recognition, tracking and machine learning algorithms. This survey aims to detect and characterize a sudden event, which is a subset of an abnormal event in several video surveillance applications. This paper discusses the following in detail: (1) the importance of a sudden event over a general anomalous event; (2) frameworks used in sudden event recognition; (3) the requirements and comparative studies of a sudden event recognition system and (4) various decision-making approaches for sudden event recognition. The advantages and drawbacks of using 3D images from multiple cameras for real-time application are also discussed. The paper concludes with suggestions for future research directions in sudden event recognition.

## 1. Introduction

Event recognition is a very significant research topic and is heavily used in many high-level computer vision applications, such as security surveillance, human-computer interaction, automatic indexing and retrieval and video browsing. With regard to security-related event recognition, a video surveillance system is readily available in most areas, including smart homes, parking areas, hospitals and community places.

The importance of surveillance systems and alert networks is to provide an immediate response during an emergency situation [1, 2]. An emergency situation is a situation that typically involves an immediate threat, which can occur at any time and place, due to multiple factors, such as a fire, medical emergency, gas leak, bomb and physical threat. Therefore, the emergency response must ensure the safety of the people and protect the emergency scene.

Event detection has become an important research area in which many researchers have focused on classifying the event as either a normal or abnormal event. Consequently, abnormal event recognition becomes a necessity in surveillance systems to ensure safety and comfort. An abnormal event may or may not be a sudden event. An example of an abnormal event that is not sudden is the case of suspicious behavior detection. An example is loitering activity that can be recognized only after a certain period of time and that does not require a split-second decision to disperse the loiterers. In contrast, an abnormal event that is recognized as sudden, e.g., in cases that involve a sudden fall in a patient monitoring system or a snatch theft, will require immediate mitigation to alert the relevant authorities.

**Figure 1.**
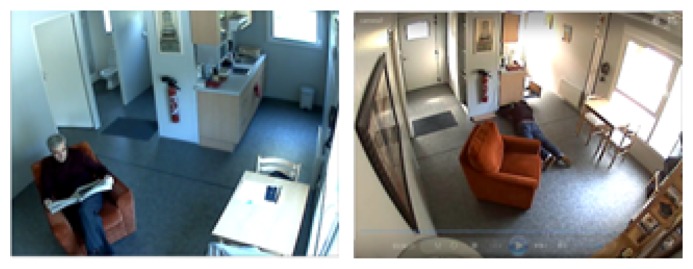
Images from the GERHOME Laboratory. Reproduced with permission from www-sop.inria.fr/stars/projects/Gerhome/Videos/ (on 20 February 2013)

In addition, sudden event recognition is becoming an important mechanism in elderly care monitoring systems to ensure their safety, security and comfort. These systems are meant to detect sudden anomalous events to reduce the risks that endanger the elderly. Moreover, the world population is expected to reach 9.3 billion by 2050 [3], and people who are older than 60 years will constitute 28% of the population. This situation requires massive resources to support the ever-increasing living cost, where human life expectancy will reach 81 years old by 2100. Senior groups can live independently if their minimum safety, security and comfort can be guaranteed. For example, the GERHOME (Gerontology at Home) [4] system, which is a pilot project that aims to provide a surveillance system for the elderly, is shown in Figure 1. The smart alert function in the GERHOME system enables the elderly to live independently and, thus, helps them to reduce the cost of care. GERHOME is equipped with a function that acts as a communication medium during an emergency situation to alert the relevant authorities.

**Figure 2.**
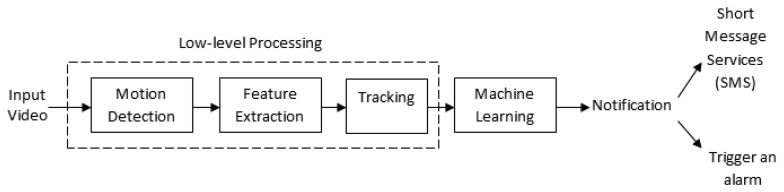
Common structure of video-based sudden event recognition system.

A summary of event recognition related review papers that involve various human activities or actions is provided in Table 1. These papers highlight the typical framework that is used in visual surveillance systems, which comprise detection, feature extraction and classification. In contrast to these papers, our review is aimed at sudden event recognition systems. Sudden event recognition is a subset of abnormal event recognition that requires instant mitigation. Because of the rapid development in event recognition systems [5–7], our survey focuses on low-level processing aspects of sudden event recognition. Figure 2 depicts the overall structure of video-based sudden event recognition, which is presented in this survey paper. Initially, the accuracy of most event detection, either sudden or non-sudden, depends on the effectiveness of the low-level processing, which involves motion detection, object recognition and tracking techniques. The extracted low-level information is then used to design a sudden event recognition system, and finally, a machine learning approach is used to recognize the sudden event.

The remainder of this paper is organized as follows: The definition of a sudden event is discussed in Section 2. Section 3 describes various types of sudden events, and Section 4 provides an in-depth explanation of existing methodologies that are related to sudden event recognition. This section also discusses machine learning techniques that can be used in sudden event recognition. Finally, Section 5 summarizes the overall content of the paper and proposes several approaches for future work.

## 2. Terms and Definitions

### 2.1. Terminology

The term “event” has been used interchangeably with the terms “action” and “activity”. No consensus on the exact definition of such terms has been made to date. Bobick [22] attempted to differentiate “action” from “activity” based on the occurrences of the events (movements and interactions), in which he has defined an “action” as a higher semantic level than an “activity”. In contrast, Govindaraju *et al.* [23] defined an “action” as an atomic motion-pattern that is often gesture-like and has a single-cut trajectory (e.g., sit or wave arm), whereas an “activity” is a series of actions in an ordered sequence that is dependent on motion patterns. These definitions are in agreement with those of Lavee *et al.* [17], who referred to an “action” as a “sub-event”, which is part of an “event”.

Event terminology is typically dependent on the nature of the application, which results in different descriptions of the event, while retaining the same aim to discover “what is happening”. Nagel [24] described events as a hierarchy of occurrences in a video sequence, whereas Hongeng *et al.* [25] defined events as several actions that occur in a linear time sequence. Candamo *et al.* [19] stated that an event is a single low-level spatiotemporal entity that cannot be decomposed further. Therefore, an “event” is used to determine the following: (1) the action being performed; (2) the actor performing the action; and (3) the objective of the action.

**Figure 3.**
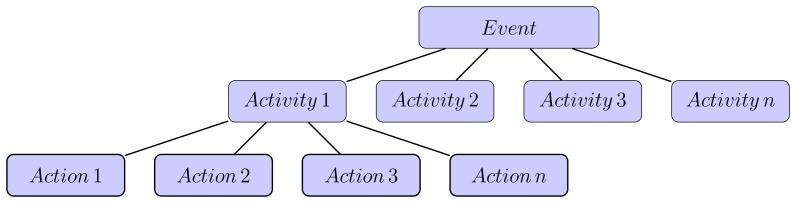
Semantic hierarchy level description.

Figure 3 shows the relationships of the levels in the semantic hierarchy, which is composed of “event”, “action” and “activity”. These terminologies were adopted from Govindaraju *et al.* [23], who stated that “event” is the highest level of the hierarchy, followed by “activity” and “action” occurrences in a video. These definitions are also in line with the description by Nagel [24], who put “event” at the top of the semantic level relationships.

### 2.2. Abnormal Event

According to Popoola and Wang [21], previous surveys focused only on abnormal event detection and did not attempt to distinguish various types of abnormal events. An abnormal event is typically assumed to be similar to the terms unusual, rare, atypical, surprising, suspicious, anomalous, irregular and outlying. Differences in opinion might be caused by the different subjective viewpoints of different studies. Xiang and Gong [26] defined an unusual event as an abnormal behavior pattern that is not represented by a sufficient number of samples during data set training, but remains within the constraints of abnormal behavior. Similarly, Hamid *et al.* [27] defined an abnormal event as a rare or dissimilar event that deviates from normal occurrences.

Based on the World Dictionary of the American Language [28], the words “sudden” and “abnormal” can be differentiated by “unforeseen” and “deviating from general rule”, respectively. Odobez *et al.* [29] simply defined an abnormal event as an “action that is performed at an unusual location and that occurred at an unusual time”. This definition can be related to the concept of Popoola and Wang [21], who considered anomalies to be temporal or spatial outliers. Therefore, in the current study, an “abnormal event” is defined as an event that is dependent on temporal and spatial information and does not conform to any learned motion patterns.

### 2.3. Sudden Event

**Figure 4.**
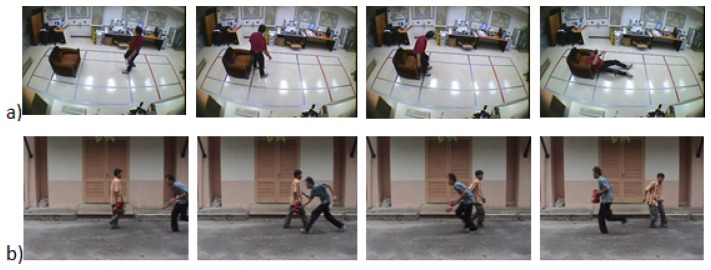
(**a**) Sudden fall. Reproduced with permission from www.iro.umontreal.ca/labim-age/Dataset/(accessed on 20 February 2013) [31]; and (**b**) snatch theft. Adapted from [32].

To date, no consensus has been reached on the definition of “sudden event”. From a psychologist's perspective, Jagacinski *et al.* [30] described a sudden event as a motion that caused significant change in the patterns of the motion trajectories. The resulting dynamic state of the object, such as direction and speed of motion, are caused by a change in the force applied to the object during the activity. As such, we define “sudden event” terminology as an abnormal event that occurs unexpectedly, abruptly and unintentionally, such that the state of the object deviates from the previous state, which invokes an emergency situation that requires fast responses; for example, a sudden fall among the elderly that might occur due to a loss of balance, a loss of support from an external object (e.g., a walker as a typical Parkinson support) or slipping after a sudden bump on a certain object. In Figure 4a, a sudden event has occurred in which the actor fell down, whereas the real intention is to sit down. Such an event implies that the actor does not follow the normal trajectory pattern. Therefore, it is important to detect this type of event, especially for an elderly monitoring system that demands an immediate mitigation action to reduce the negative consequence on them. Moreover, a sudden event happened unintentionally and does not require a built up event. For instance, a burglary is not considered a sudden event, since the thief will walk around the jewelery store with the intention to break in. Thus, there is a precedence event, which is defined as intentional, that does not comply with a sudden event definition.

Other than elderly or patient care, sudden event recognition can also be implemented on a public road or accident prone area. In an anomalous visual scene, the difference between a non-sudden and sudden event is that the former event requires a relatively longer observation scene, whereas the latter event typically takes only a few frames to be identified. For example, the case of abandoned luggage can be viewed as an abnormal event when the actor moves a few meters away from his bag and leaves it unattended for a certain period of time. Such an event is not considered a sudden event, because the event took a longer reaction time to be recognized. However, the case of a snatch theft in Figure 4b, which is detected in a public area, will be considered as a sudden event. A snatch theft event attracts the attention of the public, and it requires an urgent reaction to help the victim. The understanding of contextual information in a scene is very significant in developing an automatic sudden event recognition system. In the future, the system should be able to anticipate the same event by learning the risk factors to mitigate the crime effect.

In the case of crossing the line of a prohibited area, although it invokes an emergency situation, it is not considered as a sudden event, because it does not happen abruptly. Normally, it is an intended event, where a person intentionally crosses the alert line, as, for instance, the yellow line in a subway station or the safety line in a construction site or restricted area. However, it may be viewed as a sudden event if someone accidentally pushes a person beyond the alert line. Similarly, for a toddler monitoring system, a jump and run action is not considered as a sudden event. Although it is an abrupt event that can be detected within a few frames, it does not invoke an emergency warning, since they are just playing.

**Figure 5.**
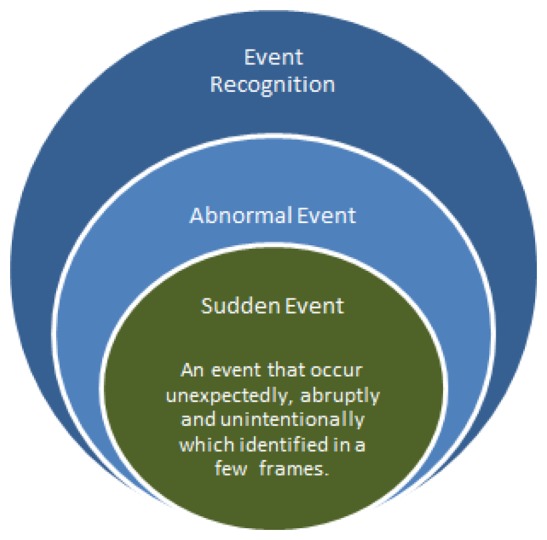
Diagram of sudden event recognition.

Therefore, as shown in Figure 5, we summarize a sudden event as: (1) a subset of an abnormal event; (2) an event that occurred unexpectedly, abruptly and unintentionally that invokes an emergency situation; and (3) an event that is detected in a few numbers of frames, which requires a fast response to mitigate the posterior risks. Table 2 summarizes several abnormal events subject to the definitions of a sudden event, such that a sudden event is recognized if all three aforementioned criteria are fulfilled.

## 3. Sudden Event Description

The awareness of the need to provide a safe and comfortable living environment has led to a surge of video-based research activity in the surveillance community. The advantage of having a video-based system is the introduction of an autonomous system that reduces the reliance on the human ability to monitor and detect anomalous events. In general, sudden event recognition for visual applications can be divided into two categories, namely, those that involve a human-centered sudden event, a vehicle-centered sudden event and a place-centered one, as explained in the following scenarios.

### 3.1. Human-Centered

**Figure 6.**
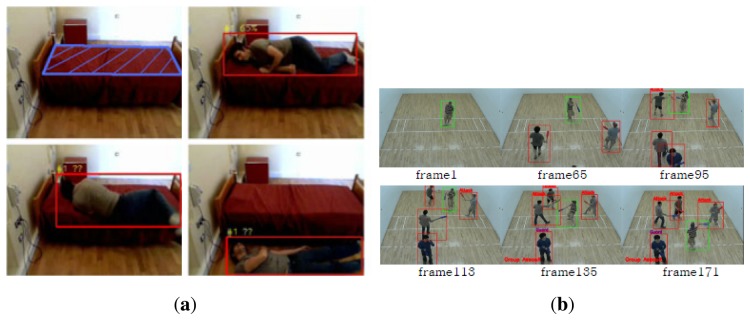
Example of a human-centered sudden event. (**a**) Patient falls out of bed. ©2011 IEEE. Reprinted, with permission, from [33]; (**b**) group interaction shows the sequence of a sudden assault. Reproduced with permission from [34]. With kind permission from Springer Science and Business Media.

This unexpected event can be defined by considering (1) single and (2) multiple persons. Examples for each of these groups are as follows:

1. Single person (Figure 6a)
  This event comprises behavior that involves only a single person without any interaction with another person or vehicle, e.g., a patient's sudden fall out of his bed or an elderly fall at a nursing home.
2. Multiple person interaction (Figure 6b)
  This event consists of behavior that involves multiple persons interacting with each other, e.g., people who fight and disperse suddenly, muggers and a snatch theft.

### 3.2. Vehicle-Centered (Figure 7)

This category of sudden event consists of behavior that is defined through human interaction with vehicles. A vehicle-centered sudden event could occur when there is an unexpected behavior suddenly on the road, such as a vehicle that deviates from the normal lane that could lead to a collision, a sudden stop in the middle of traffic and a road accident. These examples need an urgent response from the traffic monitoring system to prevent the occurrence of a serious incident.

### 3.3. Small Area-Centered (Figure 8)

This category of sudden event mainly happened due to tight spatial constraint, which may cause some inconvenience or accident, such as in an elevator cage, staircase or corridor. The small area-centered event is represented by space volume properties that include perspective and size. The characteristics of the small area-centered event are highly correlated with the semantic definitions of the sudden event. A staircase fall can occur when a person misses a step at some point, either by completely overstepping or slipping off. The risk of a sudden event happening is higher when the small area is crowded with people, such as at public stairways or escalators [37, 38]. Furthermore, the enclosed and isolated area, such as an elevator cage or a lift, might instigate a sudden assault [39] and rape.

## 4. Methods for Sudden Event Recognition

Most of the existing research on sudden event detection and recognition has been designed only to solve specific problems, and the approaches used are developed based on models and prior assumptions. The effectiveness and limitations of the existing methods are discussed below.

### 4.1. Single Person

In the case of a patient monitoring and home care assistance system, a sudden event refers specifically to a sudden fall by a patient or elderly person. Detection and tracking of the human body are useful features for early indication of a sudden fall event. However, the comfort and privacy of the person being monitored must be considered prior to system installation.

### 4.1.1. Sudden Fall

A single person's sudden fall is defined as an instantaneous change in the human body from a higher to a lower position, which is a major challenge in the public health care domain, especially for the elderly. A reliable video surveillance system, such as a patient monitoring system, is a necessity to mitigate the effect of the fall. Sudden fall detection using computer vision has been widely investigated, especially for a 2D vision system, which requires only one uncalibrated camera. A vision-based system is also preferable over other sensors, because of its non-intrusive nature. In most of the computer vision literature, low level processing can be represented by motion segmentation and geometrical feature extraction, such as the shape and posture.

A background subtraction method is typically used to extract the human silhouette to detect a fall [31, 40-44], where it is used to extract regions of interest by comparing the current frame information with the background model. This approach is a commonly used method for moving object detection. A pixel is recognized as foreground when the difference between two successive frames is significant. The most popular algorithm, which was proposed by Stauffer and Grimson [45], is based on statistical background subtraction, which employs a combination of Gaussian models to observe the probability of detecting a background pixel, *x*, at time *t*, as follows:

(1) $$P(x_{t})=\sum_{i=1}^{K}\omega_{i,t}*\eta (x_{t},\mu_{i,t},\sigma_{i,t}^{2})$$

where K is the number of distributions, which is normally set to [3, 5], *ω_i,t_* and *μ_i,t_* are the weighted and mean of the Gaussian distributions, respectively, $\sigma_{i,t}^{2}$ is the covariance matrix and *η* is the Gaussian probability density function. The Gaussian mixture is a stable real-time outdoor tracker and works well in various environments, such as variations in lighting, repetitive motion caused by clutter and long-term scene variation. The Gaussian mixture has been used successfully as a motion detector for a hierarchical multiple hypothesis tracker (MHT) [46], which can solve merge, split, occlusion and fragment problems.

Instead of using a combination of Gaussian distributions, Harish *et al.* [47] implemented a symmetric alpha-stable distribution to detect clutter background. The background appearance is characterized by spectral, spatial and temporal features at each pixel. Martel *et al.* [48] incorporated cast shadow removal into their pixel-based Gaussian mixture for surveillance application. Young *et al.* [49] combined a Gaussian mixture and weight subtraction methods between the consecutive frames to extract the foreground object. In addition, Yan *et al.* [50] fused both spatial and temporal information to the conventional Gaussian mixture using a regional growth approach. Their method can adapt to both sudden and gradual illumination changes in various complex scenes for real-time applications. However, the Gaussian approach lacks flexibility when addressing a dynamic background. Although robust background subtraction is typically computationally expensive, a different method to improve the standard algorithm is required to construct online detection [51] and an adaptive background model [42] for sudden event recognition. Most of the recent background subtraction methods focus on improving the modeling of statistical behavior [52, 53] and on using an integrated approach that includes multiple models [40, 54–56].

Several existing methods for detecting a fall that have a good detection rate are based on the fall characteristics of the human body shape, which varies drastically from a vertical to horizontal orientation during a sudden fall event. The most common geometrical features that are used in sudden event recognition are the rectangular bounding box [40, 42, 43, 57] and an approximated ellipse [58, 59]. The extracted geometrical properties include the horizontal and vertical gradients, aspect ratio, centroid, angle, projection of the head position and velocity. In [40, 57], a fall was confirmed if the aspect ratio was high, whereas [42] detected a fall if the angle between the long axis of the bounding box and the horizontal direction had a value of less than 45 degrees. However, these methods are invalid in the case of a person who falls toward the camera. Rougier *et al.* [60] used shape context matching based on a human deformation measure of a mean matching cost and the Procustes distance to detect any change in the body shape. This method is robust to small movements that occurred immediately after the fall. Wu *et al.* [61] demonstrated velocity profile features to distinguish from standing and tripping in fall event detection. The change in magnitudes characterized the horizontal and vertical velocities, which are sensitive to the camera view, especially when the person is close to the camera. Thus, their study concluded that the aspect ratio of the person's bounding box varied according to the velocity changes.

Furthermore, the use of a wall-mounted camera [31] and omni-camera [43] allowed for the monitoring of fall incidents with a wide angle view of a living room or bedroom. The motion pattern of an extracted geometrical region is particularly useful in detecting a sudden fall. Fall detection based on global and local motion clustering in [62] utilized three features, including the duration of the sudden fall, the rate of change of the human centroid and the vertical histogram projection of the human body. However, the direction of the body movement between various camera views and a changeable video frame rate would certainly affect the feature values. In [58], a motion history image (MHI) was combined with shape variations to identify the fall event. Recent motion patterns were used by [63] to detect a slip or fall event; here, a spatiotemporal motion energy (ISTE) map is employed. Additionally, Olivieri *et al.* [64] presented an extended MHI called a Motion Vector Flow Instance (MVFI) template, which extracted the dense optical flow of human actions. Although MHI retained the information for the entire sequence of images, the new information was not truly useful for recognizing a sudden event when occlusion occurred. In addition, the MVFI method characterized a fall event based on a spatio-temporal motion template that discriminates human motion effectively if the velocity information is made available.

Posture is the other important feature that is utilized in sudden fall detection; it is used to represent the different body poses. Cucchiara *et al.* [41] proposed a method that utilized a posture map with a histogram projection to monitor the movement of a person to detect a fall event. Brulin *et al.* [33] proposed a posture recognition approach in a home care monitoring system for the elderly that used principal component analysis (PCA). PCA was used to compute the principal axis of the human body and to extract the center of the human silhouette, which signified the posture center of gravity. Miao *et al.* [65] employed ellipse fitting and histogram projection along the axes of the ellipse to discriminate different types of human postures. These posture-based methods [33, 41, 65] were highly dependent on the effectiveness of the background subtraction method and were used to differentiate several similar events, such as lying down, bending and falling.

Instead of analyzing the human body as a whole, the detection of body parts, *i.e.*, head detection, has been applied in sudden fall event detection. In [59], a histogram projection of the head position was used to detect a fall incident, because the head produced the largest movement during a fall. Hazeldoff *et al.* [44] estimated the head position using a Gaussian skin color model and found a match for skin colored blobs that were close to the head. Jansen and Deklerck [66] used 3D head motion-based analysis in which a fall was confirmed when the period of vertical motion was shorter than the horizontal motion. Rougier *et al.* [67] used a 3D ellipsoid for a bounding box fitting of the head in a 2D image. A particle filer was then used to extract the 3D trajectory of the head based on the 3D velocity features for fall detection.

An event monitoring system typically involves supervised training. The extracted feature vectors are fed to the classifier, where a new scenario will invoke the system to learn it. Several machine learning methods have been used to detect a sudden fall. Rougier *et al.* [60] used a Gaussian mixture model (GMM) to classify a fall incident based on a measure of human shape deformation, whereas Faroughi *et al.* [59] used the extracted motion features to train a multi-layer perceptron (MLP) neural network. In another work by Faroughi *et al.* [68], support vector machine (SVM) was used to detect a fall based on the shape variation of the ellipse that encapsulates the silhouette and head pose. Thome *et al.* [69] developed a Hierarchical Hidden Markov Model (HHMM) that has two layers of motion modeling; Brulin [33] and Juang [70] further analyzed the features via a fuzzy inference model; whereas Tao [40] and Liao *et al.* [63] used a Bayesian inference model to detect a sudden fall. Although most of the selected machine learning methods can classify a sudden fall effectively, our review found that they have some limitations. For instance, in the fuzzy learning model developed by Juang [70], the membership function and fuzzy rules should be adapted and refined first, considering the posture variation of the elderly and young people. Moreover, the efficiency of a sudden event recognition system will be limited when generalizing fuzzy inference based on an articulated human model, because a longer response time is required. Table 3 summarizes the features that are used in the sudden fall detection systems from the selected literature.

### 4.2. Multiple Person Interaction

A multiple person interaction of a sudden event recognition system has been motivated by the public demand for a safe and secure environment. Security and surveillance systems are crucial tools in crime prevention, because they alert the required authorities to be fully aware of the threats, allowing them to mitigate the risks and consequences. Examples of sudden events that could involve multiple person interactions are snatch theft and sudden assault.

#### 4.2.1. Snatch Theft

An automatic detection system for a sudden event involving a snatch theft crime would require both the victim and thief to be identified. The snatch thief will typically approach the victim from behind. The methods proposed by [72, 73] are based on motion cues for extracting low-level motion patterns in the scene. For each video clip, the optical flow was computed, and the motion vector flow was extracted and later used to detect the snatch event. The optical flow technique is built on the assumption that the neighboring points of most pixels in an image have approximately the same brightness level. The foundation of the optical flow can be attributed to the gradient-based algorithm that estimates pixel movement between successive frames through feature similarities, which is an approach that was first proposed by Shi and Tomasi [74]. The combination of the Lucas-Kanade [75] optical flow and the Gaussian background model has yielded good foreground segmentation, and the basic optical flow can be written as follows:

(2) $$\nabla I\cdot \upsilon_{m}+\frac{\delta I}{\delta t}=0$$

where $\nabla I={\left[\frac{\delta I}{\delta x},\frac{\delta I}{\delta y}\right]}^{T}$ is the spatial intensity gradient vector. Assuming that *I*(*x,y,t*) is the intensity of pixel *m*(*x, y*) at time t, then the velocity vector of pixel *m*(*x,y*) can be denoted as follows: *v_m_* = [*v_x_*, *v_y_*]. The Lucas-Kanade method is suitable in cases where the video contains a crowded scene, because the detection operates on a single pixel basis, in which partially occluded objects can still obtain their matching features. The combination of the Lucas-Kanade approach and Gaussian-based background modeling yields a good optical flow field between two adjacent frames. The median value and the Gaussian filter are then used to reduce the noise. A predefined threshold value is necessary to determine the cutoff value and extract the label from the Gaussian background model. The joint areas between the optical flows and foreground outputs are extracted to obtain the optimal foreground image.

In [73], three motion characteristics are determined from the video stream: (1) the distance between objects; (2) the moving velocity of objects; and (3) the area of the objects. The average velocity obtained from the motion vector flow is used to analyze a sudden change in the target velocity and moving direction. During the monitoring process, more attention is required when the distance between two moving objects is decreasing. Then, the extracted feature vectors are classified using the nearest neighbor classifier.

Similarly, [72] demonstrated that the optical flow motion vector is consistent during a normal interaction and is distracted if a snatching activity has occurred. Then, a support vector machine (SVM) is used to classify between snatch and non-snatch activity. The advantage of the optical flow approach is that the motion vector is apparent during crowded scenes, where a pixel-based detection can distinguish the foreground objects and provide good results for video surveillance applications with multiple and synchronized cameras. However, the computational burden of optical flow is very high and it is very sensitive to noise and illumination changes; thus, it requires specialized hardware for real-time implementation.

In [76], Voronoi diagrams were used to quantify the sociological concept of personal space. The approximate area that has possible threatening activities is up to 0.5 meters. The temporal aspect of the Voronoi diagrams is used to identify groups in the scene, whereas a spatial area within each individual's field of view is used to classify the groups as intentional or unintentional. The tracking of each individual within this area is necessary, although it might be difficult in densely crowded scenes. Liu and Chua [77] used motion trajectories to simulate the activity of multi-agent snatch thefts. Motion trajectory patterns were used to detect the position and classify the state of an object of interest. Therefore, the extracted individual motion trajectories represent the role of each agent. The state of an object is observed and predicted based on the common pattern of using trajectory clustering. The cluster of object-centered motion patterns is modeled by the motion time series of the object trajectories. The re-occurrence of a trajectory is typically considered a normal event, whereas a rare trajectory pattern that is beyond the learning pattern is considered a sudden case. In [77], the pre-defined activity models were classified using an HMM.

Instead of performing snatching activities based on a detection or tracking method, which efficiently recognizes primitive actions, such as walking and running, contextual information [78] is used to reduce the complexity of the scene modeling. The contextual information is composed of integrated features and the event models developed from prior knowledge of object interactions between actions, poses and objects in the scene. This information will reduce the size of the possible sets, whereas it will only allow the events that can fulfill the context. A sudden change of behavior in the scene is typically derived based on the misclassified detection model, where it is quantified by using deviation measures between the observation and predefined normal behaviors. Jeong and Yang [79] presented each target activity as ground, weighted and undirected trees. Snatching activity is defined in natural language and the Horn clause. For example, a snatch event is defined by a description of (1) what the thief does to snatch something (*follow*); and (2) the thief taking someone else's belongings using force (*attack*). These two expressions, *follow* and *attack*, are detected through several primitive actions, such as running, walking, approach and turning. Then, the rules of each activity and the probabilities of the actions are learned using a Markov logic network. Therefore, the knowledge of the relationship between the people, contextual objects and events are presented as semantic information that help achieve an efficient decision-making process in recognizing the event. The contextual approach in sudden event recognition has two main advantages: (1) the training sets are composed of a limited scenario of normal behaviors only; and (2) sudden events are detected when unexpected behavior patterns are observed based on contextual features. In addition, an online clustering algorithm can be employed for the continuous video stream to detect any sudden event in a smart surveillance system.

#### 4.2.2. Sudden Assault

A crowd monitoring system helps to detect and prevent any crime cases or deliberate wrongful acts (e.g., muggers, assaults and fights) by monitoring both crowd and individual behaviors. A sudden assault indicates a situation in which one person approaches and attempts to hurt another person who was acting peacefully. Then, a fighting interaction follows after the assault. Sudden assault attacks can be observed when one blob becomes too close to another static or moving blob.

In most of the literature, the multiple person interaction process consists of segmentation, blob detection and tracking. For example, assaults that are followed by a fighting interaction are defined by blob centroids merging and splitting with fast changes in the blob characteristics. Blobs are extracted in each frame by using a background subtraction method. The blobs that represent the semantic entities are tracked by matching those blobs in consecutive frames. Object tracking has typically been performed by predicting the position in the current frame from the observation in the previous frame. One of the popular tracking methods is mean-shift tracking. Comaniciu and Meer [80] used mean-shift techniques to search for an object location by comparing the histogram properties between object *Q* and the predicted object location, *P*. Then, the similarity measure is defined using Bhattacharyya distances, $\sum_{u=1}^{b}P(u)Q(u)$, where *b* is the number of bins. This algorithm requires five or six iterations for each frame before the mean-shift converges to a single location. The highest weight obtained from the histogram similarity determines the closeness of the new location to the centroid of the tracked object. Evidently, a mean-shift tracker exhibits many advantages, such as having a faster processing speed and being more adaptable to complex scene variations [81, 82]. Mean-shift has also been enhanced by using a kernel method [83] to filter the background information.

In the case of multiple-object interactions, the interaction between an object and the background is important, because multiple objects can merge, split and occlude one another [84]. The objects can also appear, stop moving and leave the camera's field of view at any time. Some examples of multiple-object tracking algorithms include the particle filter [85], HMM filter [86], joint probability data association filter (JPDAF) [87], probability hypothesis density filter [88] and MHT [89]. However, sudden assault detection is considered a high level activity that requires efficacy in the low-level processing to determine the overall success of the detection. Several projects, such as Multi-camera Human Action Video (MuHAVi) [90], Computer-assisted Prescreening of Video Streams or Unusual Activities (BEHAVE) [91] and Context-Aware Vision using Image-based Active Recognition (CAVIAR) [92], have been conducted to act as the benchmark dataset for sudden assault behaviors, such as shot gun collapse, punch, kick, fight and run away.

Initially the actions are detected from low-level processing, such as the object motion speed and tracking of the object trajectory. At the same time, high level processing presents context language to detect actions of interest. The high-level processing presented in [79] addresses contextual information by learning predefined rules that were used to detect the occurrence of a sudden assault. For example, a fight is detected when two persons hit, kick or punch each other. These actions define a formula that has a sudden assault event term as the person is being attacked before a fight. The weights indicate how significantly the respective formula infers the likelihood of an attack or fight. Then, a ground network is constructed using Markov logic networks (MLNs) with primitive actions at the bottom level and activities at the top level. The probability of sudden event occurrences is given by the likelihood of the ground activity at the root.

The object interactions interpret the object behavior under various conditions (e.g., object orientation and proximity to detect the grouping of objects). Previously, Allen [93] demonstrated the temporal organization of events and activities in terms of first-order logic. Based on Allen temporal logic, the relationship between two events is calculated as follows:

(3) E→s{R}[P]

where *E* is the event representation of the low-level features (the set of primitives) that serve as terminals in a stochastic grammar system, s denotes a sub-event of *E*, *R* is the temporal matrix and *P* is the conditional probability output. Allen's temporal logic has been adopted to represent activities in temporal structures [94]. However, the above description is limited to recognizing activities that have complex structures and lack the difficulty that arises from noisy observations and the stochastic nature of the sub-events. Therefore, grammar-based detection has been designed to overcome the above-mentioned problems. Extensive work has been conducted on indoor and outdoor activities, with single and multi-agent events to validate the robustness of the stochastic context-free grammar (CFG) in learning the object interactions in the scene [95]. CFG typically attempts to interpret actions in terms of temporal state transitions and conditions. Ryoo [96] presented a CFG-based representation scheme as a formal syntax for representing composite and continuing activities. The system is able to recognize recursive activities, such as assault and fighting. A hierarchical process of sudden assault activity involves the descriptions and relations between sub-events, such as *standing*, *watching*, *approaching*, *attacking*, *fighting* and *guarding*. The approach can recognize continued and crowd or group activities. Although the rule induction technique provides a high-level expression of the observed scene, it is limited to rigid event attributes, which are not robust to view changes. These event attributes must be specified by an expert in advance.

Grammar requires the training of classifiers, such as HMMs [94]. Bayesian networks can detect the proximity of an agent and determine an individual person's behavior. However, using such machine learning techniques suffers from drawbacks that are related to the classifier itself and the activity in question. Each classifier typically has its own peculiarities and weaknesses. For example, HMMs have a highly sequential nature and cannot capture parallel and sub-events; thus, the scarcity of standard labeled data sets that can be employed for reliable training is a major disadvantage. In addition, the high-dimensional feature space that is associated with extremely variable activities, such as fighting, makes this task even more difficult. Another machine learning method that is used to recognize interactions between multiple persons with a grammar-based framework is the dynamic probabilistics networks (DBNs) [97] and MLN [79]. Table 4 presents the features that are used in the literature that are related to sudden event recognition for multiple person interactions.

### 4.3. Vehicle-Centered System

Sudden traffic incident detection is modeled using events, activities and behaviors among vehicles and humans. The decision to detect a sudden event depends on the inter-relation of spatio-temporal information. Furthermore, the temporal uncertainties of event occurrences are important features for sudden event detection in traffic monitoring systems.

#### 4.3.1. Person with Vehicle Interaction

Most existing traffic monitoring systems are based on motion trajectory analysis. Motion trajectory patterns can be used to detect the position and classify the state of an object of interest. Tracking trajectory approaches are based on a Kalman filter [100, 101], optical flow [102] or particle filter [103]. Isard and Blake [85] introduced the particle filter (also known as a condensation method) that exploits the dynamical information in the image sequence. This technique provides a high degree of robustness to a non-linear, non-Gaussian model, particularly in complicated scenes. In contrast to the Kalman filter [104], which assumes that the tracked object dynamic and measurement model is linear, the particle filter allows for nonlinear movement through Monte Carlo sampling. Let *X_t_* and *Z_t_* be the target state and observation in each frame, respectively, and the conditional state density, *p*(*X_t_* | *Z_t_*), is represented by $s_{t}^{\left(n\right)}:n=1\ldots ,N$. Then, the posterior distribution is obtained through a set of particles, which can be approximated as follows: (*s*^(*n*)^, *π*^(*n*)^ | *n*) = 1…,*N*. Each particle, *s*^(*n*)^, is the target model that specifies the particle location, (*x*, *y*), velocity, (*x̂*, *ŷ*), and size, (*H_x_*, *H_y_*). The scaling change, *â*, is determined by the weights, $\pi_{t}^{\left(n\right)}$. In [103], the particle filter can effectively complete lane detection and tracking in complicated or variable lane environments that include highways and ordinary roads, as well as straight and curved lanes, uphill and downhill lanes and lane changes.

Other common tracking trajectory approaches include dynamic programming [105], the adaptive Gaussian mixture model [106] and HMMs [107, 108]. These trajectories are used to profile the actions of a target, which is tracked to recognize any sudden event automatically. Then, the motion trajectory patterns are commonly learned using the HMM [109, 110], expectation-maximization (EM) [107], fuzzy models [111] and statistical methods [102, 112]. An HMM learning trajectory is performed on a new unknown video by using known normal events. For the unseen object trajectory, *i*, the likelihood of observing *i* given any HMM of normal events, *m_k_*, is denoted by *L*(*i* | m*_k_*). If the maximum likelihood is less than a threshold, *i.e.*,

(4) $$\max_{k}\{L(i|m_{k})\}<Th_{A}$$,

where *Th_A_* denotes the threshold and trajectory *i* is detected as sudden. HMM-based methods are robust when they learn various event behavior patterns for sudden traffic incidents, such as sudden stops, reckless driving, illegal lane driving or accidents. Meanwhile, the EM algorithm can guarantee a fast convergence time, and the model was designed by simply performing on local maximum data.

Another approach to analyzing motion patterns is using a stochastic model [113] or statistical methods that attempt to calculate the probability of an abnormal event in a video scene [102]. In [113], a traffic flow is modeled to detect sudden incident behaviors of the vehicles at intersections. The system records the historical motion of vehicles to build a stochastic graph model based on a Markovian approach. A sudden incident is detected when the current motion pattern is not recognized or any specific sequence of tested video cannot be parsed with the stochastic model. The low complexity and flexibility of the binary coding of the historical motion and the stochastic model approach are reliable for use in real-time systems. In contrast to a statistical model [102], the occurrence of a sudden change in the object motion can be detected as early as possible, specifically when the object arrives at position *k* (the sample points in T_*_, where T_*_ denotes the trajectory of the object). Statistical motion patterns can be formulated according to the Bayes rule, where the probability of ϕ*_j_* given T_*_ can be calculated as follows:

(5) $$P(\phi_{j}|T_{*})=\frac{P(T_{*}|\phi_{j})P(\phi_{j})}{\sum_{m=1}^{C}P(T_{*}|\phi_{m})P(\phi_{m})},j=1,2,\ldots ,C$$

*P*(*ϕ_j_*) is the ratio of the number of samples of *m* observations. The highest probability at point *k* is used to predict the sudden changes in the object behavior at the current position, *k*. The Bayesian method predicts the probability of a future event by using likelihood and prior information, where the maximum *a posteriori* is usually used to select the final decision. In contrast to the Bayesian method, an HMM requires an optimized number of states, which can be reduced through sampling to limit the possible sets. However, HMMs are the most effective method for modeling temporal data by forming structural relationships among the variables in the system. The Markov approach models any unseen sequence of states as having a high probability of being detected as a sudden event. At the same time, the Bayesian approach performs well in real-time applications, but most cameras require manual calibration to extract the actual driveways from the video.

Sudden traffic incidents can also be modeled by using semantic rules [94, 114, 115], which require human interpretation of such events and are validated using the existing data. In [116], the semantic rules are designed based on the observation that a sudden change in the velocity and driving direction extracted from the motion vector could indicate an accident. Furthermore, there is a higher accident risk as the vehicle becomes close to the other vehicles. Rules-based learning is similar to the investigation of vehicle behaviors by applying algorithms with logical reasoning [114].

In addition, rules-based learning can be viewed in a CFG approach. Ivanov and Bobick [115] proposed a stochastic CFG (SCFG) and stochastic parsing model to recognize the activities in a video scene. In general, the motion trajectories of low-level image features are transformed to a set of symbols (an alphabet) based on some prior knowledge. The symbol stream is then fed into the event rule induction algorithm to extract hidden information, to recognize the behavior of the object and to distinguish the events. The SCFG can automatically learn models of outdoor activities in traffic [117], such as sudden lane changes and sudden stops. The grammar learned is extracted from a known class of activity. Meanwhile, the example selector is a search-based algorithm that automatically selects unknown activity to the grammar learner. After a few iterations, the conditional classification entropy will represent the amount of uncertainty in the classification of unknown activity. Zhang *et al.* [94] used a Minimum Description Length (MDL)-based rule induction algorithm to investigate hidden temporal structures and SCFG to model the complex temporal relations between sub-events of vehicle behaviors at traffic light events. SCFG could represent parallel relations in complex events, and the proposed multithread parsing algorithm could recognize the occurrence of sudden incidents in the given video stream.

Table 5 presents the employed features that are related to sudden event recognition for person vehicle interactions in the literature, and Table 6 presents the trends of interest in research on sudden event recognition for three main categories and the performance comparison performance against related work in the type of event detection and the event learning algorithm.

### 4.4. Multi-View Cameras

Multi-view cameras play an important role in real-time applications to cover the maximum observations of the events that take place. A wide-angle camera view allows for the detection of higher-priority event occurrence and provides more sophisticated event recognition and planning of further actions to be taken. For example, sudden event recognition has motivated researchers to apply a multi-camera system for image stream processing. This processing involves the recognition of hazardous events and behavior, such as falls [119, 120], activity recognition among multiple person interactions and person-vehicle interactions [99, 121].

Cucchiara *et al.* [119] exchanged visual data between partially overlapped cameras during camera handover to deform the human shape from people's silhouette. The video server (multi-client and multi-threaded transcoding) transmits a video stream sequence to confirm the validity of the received data. Thome *et al.* [120] used the fusion of the camera view based on a fuzzy logic context to form a multiple view pose detector for fall detection. Anderson *et al.* [118] proposed a 3D human representation that was acquired from multiple cameras, called a voxel person. Auvinet *et al.* [122] used a multi-camera network to construct the 3D shape of people and to detect falls from the volume distribution along the vertical axis. Shieh and Huang [123] implemented a fall detection algorithm in a multi-camera video system to fetch the images from the monitored region. The homography-related positions were utilized to construct a background model for each reference camera, whereas the multiview data observation measured across multiple cameras was used to track the person in the scene. The multi-camera view images improved the noise reduction and edge contour algorithm and, thus, refined good falling-like postures to alert the system.

Therefore, many studies have attempted to use a multi-view camera for a sudden fall detection system to provide 3D information. This choice was made because of the limitations of a single camera view in terms of precisely detecting a fall when a person's movement is parallel with the camera's view. Furthermore, the movement of a person perpendicular to the camera view results in an occlusion and a static centroid point with a larger size.

Another advantage of a multi-view camera is that the constructed 3D reference system could minimize the occlusion effects and enhance the tracking accuracy [123]. Several points in the human silhouette that are extracted from multiple cameras could improve the estimation of the 3D human body pose and perform object tracking with automatic initialization [121]. The advancements in the pose estimations in a 3D environment have opened doors to many surveillance applications and intelligent human-computer interactions.

However, there are always the tradeoffs between achieving 3D data when modeling the human body and the computational cost, as well as the robustness of the multi-view cameras in real-time applications. F irst, the enormous amount of information that is needed to infer 3D models requires a large amount of computational power to automate the data collection and data processing. Second, the multiple camera views will obtain features that are not unique, due to variations in the background scene. The background scene variations will affect the runtime performance and increase the computational load during the shape matching processing. Therefore, the computational complexity of the developed algorithms is incompatible with a real-time constraint. Moreover, the multi-view cameras require accurate camera calibration and additional cross-calibration steps. Table 7 summarizes the selected publications in relation to sudden event recognition using multi-view cameras.

## 5. Discussion and Future Directions

In this paper, we provide a review of sudden event recognition apart from the recognition of abnormal events; sudden event recognition has the additional structure that an abnormal event must occur without any warning (be unexpected), which causes an emergency situation that requires an immediate reaction. We describe sudden event recognition in two areas; human-centered and vehicle-centered, along with their requirements for successful detection. This section provides suggestions to extend the research to attain a potential that appears to be promising from our perspective.

First, we provide an overview of the detection methods in relation to sudden events that involve low-level processing, such as background modeling, feature extraction and tracking. We focus on the comparative study of a different algorithm that is applied to handle common issues, such as noisy and dynamic background, indoor and outdoor environments, occluded objects, initialization for tracking and the significant features that represent the occurrences of sudden events. There are a substantial number of issues that should be improved to increase the quality of the low-level processing. For example, a sudden fall algorithm can only address an event that has a single person in the scene. Further study is needed to carry out sudden fall detection with multiple people in the scene. Another important issue is to emphasize the tracking of multiple objects simultaneously.

Next, the requirements for sudden event detection in real-time system implementations are reviewed. These requirements include a suitable efficiency of the algorithm, a storage capability suitable for an online system and at reasonable computational time. Therefore, another open-ended research area should focus on early detection [134, 135] to prevent a severe incident and to gain an informative data representation to analyze the scene. Thus, high-level visual content presented in semantics event detection is used to bridge the gap between low- and high-level processing. The contextual grammar-based approaches and logic programming are examples of the high-level processing that is required in sudden event recognition. The high-level event description enhances the understanding of event, which is semantically based on the spatiotemporal features to localize and detect the event of interest. In addition, the rules-based approach that used an automatic learning of prior knowledge could reduce the cost of hiring an expert. Further studies are needed to research the usability and effectiveness of high-level event descriptions for real-time purposes. In addition to real-time applications, protecting the privacy of the person in a monitored area should be considered. Then, modeling the graphical representation [136] of human actions guarantees the comfort and safety of the user.

A multi-camera view is reviewed to support sudden event detection in a real-time implementation. An efficient real-time system that can generate alerts when sudden events occur is important as an early detection mechanism, in contrast to the current implementation of visual systems, which are mainly used to investigate an event after it has already occurred. A sudden event occurs unexpectedly and requires a fast response to mitigate the event before it becomes more severe. Therefore, reconstruction of a 3D human body or vehicles from multi-view image data is attempted to enhance current surveillance applications and human-computer interactions. The 3D data representations outperform the 2D methods in the quality of the full-volume of the human body, which is gathered from shape-silhouettes and some refinement techniques. The data captured from multi-omnidirectional cameras could improve the view of a person in noisy environments and occlusions. However, the reconstruction of 3D data has some restrictions in providing quality silhouette data in the segmentation process, due to shadows, self-occlusion and merging of body parts from multiple view image data. Thus, 3D data can be reconstructed only in a limited coverage area with a controlled number of cameras. Furthermore, although 3D reconstruction capabilities have extended the research potential of event recognition and become the main focus of many researchers, we strongly believe that 2D modeling can still be improved. The difficulties in multi-view cameras, such as calibration steps, decreased runtime performance and high computational complexity, can be reduced.

To conclude, we highlighted the methodologies that are used in sudden event recognition. In general, the learning of a sudden event is divided into two major categories, namely, object trajectory and rule-based learning algorithms. Two other important parameters are the speed and acceleration, which can be considered in the tracking process to classify objects into different classes, such as moving people and vehicles. The choice of efficient machine learning for better classification techniques depends on the significance of the extracted features in representing the events and the lacking of robustness in recognizing all types of sudden events. Statistical machine learning has also become a trend in the event classification and recognition process.

## Figures and Tables

**Figure 7.**
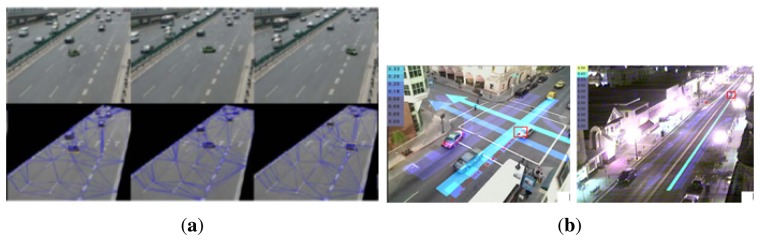
Example of vehicle-centered sudden event. (**a**) Car makes a U-turn in the middle of the road. ©2008 IEEE. Reprinted, with permission, from [35]; (**b**) a car suddenly stops and makes a U-turn. ©2011 IEEE. Reprinted, with permission, from [36].

**Figure 8.**
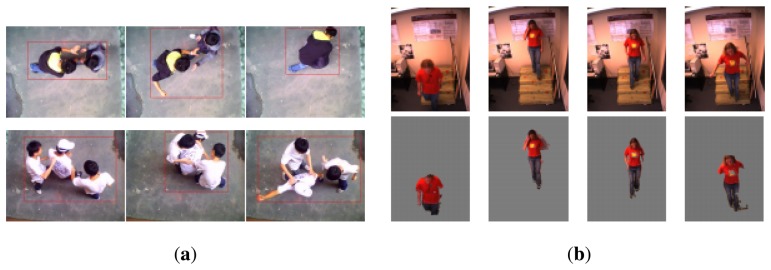
Example of a space-centered sudden event. (**a**) Elevator cage monitoring. ©2009 IEEE. Reprinted, with permission, from [39]); (**b**) staircase monitoring. ©2006 IEEE. Reprinted, with permission, from [37]).

**Table 1.** Previous related surveys.

**Year**	**First Author**	**Focus**	**Reference**
1999	Aggarwal	Human movement analysis	[8]
2002	Wang *et al.*	Human motion estimation and activity understanding	[9]
2004	Aggarwal and Park	Recognition of actions and interaction	[10]
2004	Weiming Hu *et al.*	Detection of anomalous behavior in dynamic scenes	[11]
2006	Thomas B. Moesland	Human body motion and recognition	[12]
2006	Gandhi. T	Pedestrian detection based on various cases	[7]
2007	Pantic *et al.*	Behavior understanding	[13]
2008	Morris B.T	Trajectory analysis for visual surveillance	[14]
2008	Teddy Ko	Behavior analysis in automated video surveillance	[15]
2008	Turaga *et al.*	Human activities recognition	[16]
2009	G.Lavee *et al.*	Video event understanding (abstraction and event modeling)	[17]
2009	Varun Chandola	Anomaly detection	[18]
2010	Joshua Candamo	Event recognition in transit applications	[19]
2010	Ji and Liu	Recognition of poses and actions in multiple view	[20]
2011	Popoola and Wang	Contextual abnormal human behavior	[21]

**Table 2.** Sudden event criteria.

**Example of Case**	**Sudden Event Criteria**		

	**Abrupt, Unintentionally and Unexpected**	**Short Period of Observation Scene**	**Invoke Emergency Situation/Attention That Requires Immediate Response**
Elderly/Patient walk and fall	✓	✓	✓
Snatch theft	✓	✓	✓
Crossing a prohibited area	×	✓	✓
Children jump and run	✓	✓	×
Burglary	×	×	✓
Abandoned luggage	✓	×	×

**Table 3.** Experimental results in relation to sudden fall (TP: true positives; TN: true negatives).

**Features for Event Detection**	**Results**	**Ref**
2D features: bounding box aspect ratio	Parallel and top view camera perform >80% within a distance of 4–7 m Top view camera limits the precision within a distance of 1–4 m (< 80%)	[41]
Aspect ratio, horizontal and vertical gradient	Accurately detect single person fall indoors and outdoors 100%, but <80% accuracy for a person fall with multiple people in the scene	[43]
Horizontal and vertical velocities profile	ANOVA analysis indicated that the peak horizontal and vertical velocities were higher (*p* = 0.001) than those in the normal activities	[61]
2D silhouette aspect ratio and utilized personal information information, such as weight, height and health history	The successful rates of fall detections with and without personal information are 79.8% and 68%, respectively	[44]
Bounding box aspect ratio and covariance matrix	Only preliminary results; no quantitative analysis	[40]
Vertical projection histogram of silhouette	Correct detection rate is 84.44%	[71]
Shape deformation using shape context	Reduce error rate from 9.1% to 3.8% using Procrustes distance compared to other 2D features	[60]
Motion features and metric rectification	TP = 97.6%, TN = 86.7%	[69]
Integrated spatio-temporal map and shape variations	Slip only and fall events detected with 90% and 95% accuracy, respectively;	[63]
Spatio-temporal motion from dense optical flow	Performed on six classes of action and fall events detected with 99% accuracy	[64]
Motion history image (MHI); shape variations and motion quantification	Sensitivity of 88% and an acceptable rate of false detection with a specicity of 87.5% for 24 daily activities	[58]
Global motion: period of fall, change of centroid location and vertical histogram projection	Achieve correctness ratio of about 93% with a 13% missed ratio; the ratio of FPis 0% for all 28 events	[62]
Approximated ellipse, projection histogram and temporal changes of head positions	TP = 94.3% and TN = 96.9% for 10 classes of action	[59]
Principal component analysis (PCA)	>85% fall detection rate	[57]
3D orientation of image	Only preliminary results; no quantitative analysis	[66]
Length and width ratio (DFTcoefficient) of Fourier transform histogram projection	Average recognition rate for four classes of action is 97.8%	[70]
Posture-based with point tracking	Achieve accuracy of 74.29%	[33]
Posture-based probabilistic projection maps	Average accuracy >95% in classifying human postures	[42]
Head tracking using particle filter	Reasonable mean error of 5% at five meters	[67]

**Table 4.** Experimental results in relation to sudden events with multiple person interaction. HMM, Hidden Markov Model; MLN, Markov logic network; SVM, support vector machine.

**Features for Event Detection**	**Results**	**Reference**
Motion trajectories	High recognition rate; almost 100% using HMM classifier, while the proposed model observation decomposed HMM is less sensitive	[77]
Velocity measured using optical flow	Results reported that 80% of snatching events are detected	[73]
Optical flow	Achieved accuracy of 91%	[72]
Blob detection, motion features semantics behavior representations	100% precision and 32% recall using BEHAVE dataset	[98]
Silhouette ellipse representation and convex hull with context free grammar representations,	Accuracy reported for the deterministic approach is 91.6%, while the probabilistic approach achieves 93.8%	[96]
Logic programming with context free grammar representations	Tested using four techniques; (1) Traditional MLN with Backward Induction (TBI); (2) Traditional MLN with Q-values (TQV); (3) Modified MLN with Backward Induction (MBI); and (4) Modified Q-values (MQV). Overall, MQV achieved a high precision value, almost 100%, while TQV is less sensitive	[79]
Learning context of group activities	The results reported that SVM outperformed rule-based learning with ROC > 0.81 for six different actions	[99]

**Table 5.** Experimental results in relation to a sudden event for a person with vehicle interaction (TP: true positives, TN: true negatives).

**Features for Event Detection**	**Results**	**Ref**
Spatio-temporal of Markov Random Field (MRF) for motion vectors and object tracking	Overall success rate is 94.6% for horizontal and vertical traffic	[110]
Discrete Cosine Transform (DCT) coefficient feature vector with traffic detections using Gaussian mixture HMM	HMM classifier correctly detected 91% to 94% under different illumination situations	[107]
Spatio-temporal motion trajectories using MRF	Able to detect accidents at a very high recall rate, which is more than 90%	[114]
Blob tracker using Kalman filter	Results reported that the percentage of falsely classified vehicle modes was about 3%	[100]
Extracted features: shape, position, motion and track object using Kalman filter	Correctly classify five types of vehicle behavior and pedestrians with 86% to 96% accuracy	[105]
Motion trajectories and hierarchical clustering	Half total error rate (HTER) = 11% and area under the curve (AUC) > 0.8	[109]
Motion trajectories; velocities of motion vector	The accuracy increases up to 95%, tested on two video clips (277 frame sequences)	[116]
Target trajectories with context free grammar (CFG) representations	Results reported using (1) stochastic CFG, TP = 86%, TN = 14%; (2) HMM, TP = 55%, TN = 45%	[117]
Optical flow and K-means clustering	Only visual sample results; no empirical analysis	[102]
Local motion patterns to initialize the Gaussian mixture model (GMM)	Accuracy of detection measured = 83.23%, and the error rate is 16.77%	[113]
Motion trajectories and C-means clustering	Results reported that the false rejection rate (FRR) = 6%, and the false acceptance rate (FAR) = 8.3%	[111]
Track target trajectories and learn rules using grammar representations	The accuracy increased from 73% to 98.6% when tested on five traffic sub-events with different parsing parameters, *θ* and *ω*	[94]
Lane tracking using particle swarm optimization (PSO) particle filters (PF)	Results indicated that particle filter output is smoother than the PSO-PF algorithm	[103]

**Table 6.** The trends of interest in sudden event recognition algorithms applicable in crime prevention, traffic monitoring and home care/hospital assistant system (RT: real-time implementations). MLP, multi-layer perceptron; EM, expectation-maximization; GMM, Gaussian mixture model

**Category**	**First Author**	**Yr**	**Event**	**Classifier/Matching**	**RT**	**Ref**
Crime Prevention	Liu	06	Snatching	HMM	N	[77]
	Julio Cezar	07	Attack, threatening	Voronoi diagram	Y	[76]
	Ryoo	09	Approach, attack, punch, kick	Grammar learning	N	[96]
	Goya	09	Purse snatching	Nearest neighbor classifier	N	[73]
	Jeong	10	Attack, fight, follow, snatch	Markov logic network	N	[79]
	Ibrahim	12	Snatching	SVM	N	[72]
	Zhang	12	Approach, attack, fight	SVM	N	[99]

Traffic Monitoring	Kamijo	00	Video of reckless driver, bumping accident, sudden stop and start, passing	HMM	N	[110]
	Xiaokun Li	04	Traffic behavior at the highway	HMM	Y	[107]
	Kamijo	04	Accidents detection	Logical reasoning	N	[114]
	Veeraraghavan	05	Turning, lane change, sudden stops	Trajectory analysis	Y	[100]
	Kumar	05	Vehicle behavior for accident detection	Bayesian classifier	Y	[105]
	Jiang	07	Vehicle U-turn, sudden brake and pull over the road	HMM	N	[109]
	Chen	07	Traffic behavior at intersection,	MLP and RBF	N	[116]
			car crash event			
	Veeraraghavan	09	Lane changes, sudden stop at intersection	Grammar learning	Y	[117]
	Imran	10	Traffic behavior at intersection	EM algorithm	N	[102]
	Hernandez	10	Traffic behavior at intersection	Hidden Markov Network	N	[113]
	Hsieh	11	Traffic behavior at intersection	Fuzzy SOM	N	[111]
	Zhang	11	Traffic incidents at the crossroad	Minimum Description Length (MDL)	N	[94]
	Cheng	12	Sudden lane changes	Particle Swarm Optimization (PSO)	Y	[103]

Homecare/Hospital Assistant System	Ge Wu	00	Walk, sit down, down stairs, lying down, tripping	Velocity profile	N	[61]
	Ji Tao	05	Simulated indoor standing, walking, falling down	Statistical (Hypothesis testing)	N	[40]
	Cucchiara	05	Simulated indoor walking, crouching, sitting and falling down	Bayesian classifier	N	[41]
	Miaou	06	Fall detection using omni camera view	Bounding box aspect ratio	N	[43]
	Anderson	06	Indoor walking, fallen down, kneeling and getting up	HMM	N	[57]
	Jansen	06	3D image	Learn contextual model	N	[66]
	Thome	06	Indoor video sequence of a walking-falling-lengthened motion patterns	HMM	N	[69]
	Vishakarma	07	Indoor and outdoor walk and fall; fall in crowd scene	Deterministic (gradient value and fall angle)	N	[42]
	Juang	07	Indoor walk, jogging, bend,			
			lying down and falling	Fuzzy	N	[70]
	Rougier	07	Indoor walk, bend, sit, lying down and fall	Ellipse ratio, orientation and motion quantity	N	[58]
	Lin	07	Indoor standing, squatting, fallen down	Centroid and vertical histogram projection	N	[62]
	Foroughi	08	Indoor walk, run, stumble, limp, sit, bend and lie down	MLP neural network	N	[59]
	Foroughi	08	Indoor walk, run, stumble, limp, bend, sit and lie down	SVM	N	[68]
	Hazelhoff	08	Indoor walking, bending, sitting and fall	Gaussian classifier	Y	[44]
	Anderson	09	Simulated on different types of falls	Fuzzy Logic	N	[118]
	Liu	10	Standing, sitting and fall postures	KNNclassifier	N	[71]
	Rougier	11	Indoor walk, bend, sit, lying down and fall	GMM classifier	N	[60]
	Liao	12	Indoor and outdoor walk, run, sit, stand, lying down and crouching down	Bayesian belief network	N	[63]
	Miao Yu	12	Indoor walk, sit, bend, lying on sofa and fallen down	Directed acyclic graph SVM	N	[65]
	Olivieri	12	Indoor walk, jogging, bend, lying down and falling	KNN classifier	N	[64]
	Brulin	12	Simulated indoor sitting, standing, lying, squatting and fall from bed	NN; Fuzzy Logic	N	[33]

**Table 7.** Multi-view camera implementations in relation to sudden event recognition (RT: real-time implementations).

**First Author**	**Yr**	**Methods**	**Results**	**RT**	**Ref**
Weinland	06	Motion history volumes for kick, punch, turn and get up actions	Classifier methods are PCA, Mahalanobis distance and LDAwith an average rate of 73%, 93% and 92%, respectively	N	[124]
Denman	06	Optical flow for vehicle tracking to predict velocities—four cameras	Overall Euclidean tracking error of 11.41 pixels	Y	[125]
Cuchiarra	07	Warping person silhouette from multi-camera view	Only visual; no empirical data analysis	Y	[119]
Weinland	07	3D exemplars based on HMM; silhouette projections for kick, punch, turn and get up actions	Average recognition rate for all four cameras are 81.4%	N	[126]
Taj	07	Object detection using statistical change detection and tracking using graph matching Events classifier based on HMM	Overall accuracy for a warning event is 94.45% and for an alarm event is 92.86%	N	[127]
Calderara	07	Used two cameras to detect and track using background suppression and appearance-based probabilistic approach Applied Bayesian network for motion trajectory classifier	Accuracies detected are 100% for a sudden event and 97.5% for a normal event	N	[128]
Thome	08	3D multiple view pose detector with motion modeling based on layered HMM (LHMM)	Good ability to detect sudden changes of human pose with a rate of 82% correct detection for fall events	Y	[120]
Adam	08	Statistical low-level processing; eliminate tracking based algorithm	Results recorded the detection rate as > 95%.	Y	[129]
Antonakaki	09	Videos from three cameras performed background subtraction, homography estimation and target localization	Overall precision is 98.6% using SVM and HMM for a sudden event, such as FightChase, FightRunAway, etc.	Y	[130]
Uson	09	Voxel-based algorithm combined with probabilistic models using eight cameras	Average detection rate for a fall down event is 95.6%	N	[131]
Anderson	09	Extract person profile (height, width, length) from silhouette in four cameras	Three fuzzy variable (Upright, On the groundand In-between) positions; the precision recorded was 83.1%,97.6% and 67.7%, respectively	N	[118]
Drews	10	Estimate crowd size and activity using optical flow from two cameras	HMM and Bayesian network (BN) to classified between calm, low and high movement BN outperformed HMM with less false alarms	N	[132]
Peyman	11	Traffic flow monitoring for a sudden incident at an intersection using a hybrid scale invariant feature transform (SIFT)—two cameras	The SVM learning machine performed recall above 93% and 95% precision	N	[133]
Auvinet	11	Analyzed volume distribution along vertical axis for 3D shape of people	Results achieved 99.7% sensitivity and specificity with four cameras.	Y	[122]

## References

[1] Andrew S.D. (2012). Personal emergency response systems: Communication technology aids elderly and their families. J. Appl. Gerontol..

[2] Edlich R.F., Redd J.L., Zura R.D., Tanner A.E., Walk E.E., Wu M.M. (1992). Personal emergency response systems. J. Burn Care Rehabil..

[3] (2004). World Population to 2300. Department of Economic and Social Affairs.

[4] Bremond F., Zouba N., Anfonso A., Thonnat M., Pascual E., Guerin O. (2010). Monitoring elderly activities at home. J. Gerontechnol..

[5] Vaidehi V., Ganapathy K., Mohan K., Aldrin A., Nirmal K. Video Based Automatic Fall Detection in Indoor Environment.

[6] Wang H. Vehicle Flow Measuring Based on Temporal Difference Image.

[7] Gandhi T., Trivedi M.M. Pedestrian Collision Avoidance Systems: A Survey of Computer Vision Based Recent Studies.

[8] Aggarwal J.K., Cai Q. (1999). Human motion analysis: A review. J. Comput. Vis. Image Underst..

[9] Wang L., Hu W., Tan T. (2002). Recent developments in human motion analysis. Patt. Recognit. Lett..

[10] Aggarwal J.K., Park S. Human Motion: Modeling and Recognition of Actions and Interactions.

[11] Hu W., Tan T., Wang L., Maybank S. (2004). A survey on visual surveillance of object motion and behaviors. IEEE Trans. Syst. Man Cybern. Part C Appl. Rev..

[12] Moeslund T.B., Hilton A., Kruger V. (2006). A survey of advances in vision-based human motion capture and analysis. Comput. Vis. Image Underst..

[13] Pantic M., Pentland A., Nijholt A., Huang T.S. (2007). Human Computing and Machine Understanding of Human Behavior: A Survey.

[14] Morris B.T., Trivedi M.M. (2008). A survey of vision-based trajectory learning and analysis for surveillance. IEEE Trans. Circuits Syst. Video Technol..

[15] Teddy K. A Survey on Behavior Analysis in Video Surveillance for Homeland Security Applications.

[16] Turaga K., Chellappa R., Subrahmanian V.S., Udrea O. (2008). Machine recognition of human activities: A survey. IEEE Trans. Circuits Syst. Video Technol..

[17] Lavee G., Rivlin E., Rudzsky M. (2009). Understanding video events: A survey of methods for automatic interpretation of semantic occurrences in video. IEEE Trans. Syst. Man Cybern. C.

[18] Chandola V. (2009). Anomaly detection: A survey. J. ACM Comput. Surv..

[19] Candamo J., Shreve M., Goldgof D.B., Sapper D.B., Kasturi R. (2010). Understanding transit scenes: A survey on human behavior-recognition algorithms. IEEE Trans. Intell. Transport. Syst..

[20] Ji X., Liu H. (2010). Advances in view-invariant human motion analysis: A review. IEEE Trans. Syst. Man Cybern. C.

[21] Popoola K., Wang O.P. (2012). Video-based abnormal human behavior recognition-a review. IEEE Trans. Syst. Man Cybern. C.

[22] Bobick A. (1997). Movement, activity, and action: The role of knowledge in the perception of motion. Phil. Trans. R. Soc. Lond..

[23] Venu G. A Generative Framework to Investigate the Underlying Patterns in Human Activities.

[24] Nagel H.H. (1988). From image sequences towards conceptual descriptions. Image Vis. Comput..

[25] Hongeng S., Nevatia R. Multi-agent Event Recognition.

[26] Gong X. (2008). Video behavior profiling for anomaly detection. IEEE Trans. Pattern Anal. Mach. Intell..

[27] Johnson A., Hamid R., Batta S., Bobick A., Isbell C., Coleman G. Detection and Explanation of Anomalous Activities.

[28] David B. G. (1964). Webster's New World Dictionary of The American Language.

[29] Varadarajan J., Odobez J. Topic Models for Scene Analysis and Abnormality Detection.

[30] Jagacinski R.J., Johnson W.W., Miller R.A. (1983). Quantifying the cognitive trajectories of extrapolated movements. J. Exp. Psychol. Hum. Percept. Perform..

[31] Auvinet E., Rougier C., Meunier J., St-Arnaud A., Rousseau J. (2010). Multiple cameras fall dataset.

[32] Ibrahim N., Salasiah, Lee Y.S., Mustafa M.M., Hussain A. Snatch Theft Detection using Low Level Features.

[33] Brulin D., Benezeth Y., Courtial E. (2012). Posture recognition based on fuzzy logic for home monitoring of the elderly. IEEE Trans. Inf. Technol. Biomed..

[34] Ryoo M.S., Aggarwal J.K. (2011). Stochastic representation and recognition of high-level group activities. Int. J. Comput. Vis..

[35] Hao S., Chao L., Qi W., Zhang X. Real-time Detection of Abnormal Vehicle Events with Multi-Feature over Highway Surveillance Video.

[36] Kihwan Kim, Dongryeol Lee, Essa I. Gaussian process regression flow for analysis of motion trajectories.

[37] Snoek J., Hoey J., Stewart L., Zemel R.S. Automated Detection of Unusual Events on Stairs.

[38] Fuentes L.M., Velastin S.A. (2006). People tracking in surveillance applications. Image Vis. Comput..

[39] Tang Y., Wang X., Lu H. Intelligent Video Analysis Technology for Elevator Cage Abnormality Detection in Computer Vision.

[40] Tao J., Turjo M., Wong M.F., Wang M., Tan Y.P. Fall Incidents Detection for Intelligent Video Surveillance.

[41] Cucchiara R., Grana C., Prati A., Vezzani R. (2005). Probabilistic posture classification for human-behavior analysis. IEEE Trans. Syst. Man Cybern. A.

[42] Vishwakarma V., Mandaland C., Sural S. (2007). Automatic Detection of Human Fall in Video.

[43] Miaou S.G., Sung P.H., Huang P.Y. A Customized Human Fall Detection System Using Omni-Camera Images and Personal Information.

[44] Hazelhoff L., Han J., deWith P.H.N. Video-based Fall Detection in the Home using Principal Component Analysis. SpringerLink 5259.

[45] Stauffer C., Grimson W.E.L. Adaptative Background Mixture Models for a Real-time Tracking.

[46] Zulkiefley M.A., Moran B. (2012). Robust hierarchical multiple hypothesis tracker for multiple-object tracking. Expert Syst. Appl..

[47] Harish B., Lyudmila M., Alin A. (2010). Video foreground detection based on symmetric alpha-stable mixture models. IEEE Tran. Circuits Syst. Video Technol..

[48] Martel-Brisson N. Moving Cast Shadow Detection from a Gaussian Mixture Shadow Model.

[49] Young-Sook L., Wan-Young C. (2012). Visual sensor based abnormal event detection with moving shadow removal in home healthcare applications. Sensors.

[50] Yan Q., Yang X.Y., Xiao K.K., Traversoni L. Real-time Foreground Detection Based on Tempo-spatial Consistency Validation and Gaussian Mixture Model.

[51] Zulkifley M.A., Moran B., Rawlinson D. (2012). Robust foreground detection: A fusion of masked grey world, probabilistic gradient information and extended conditional random field approach. Sensors.

[52] Tombari F., Di Stefano L., Lanza A., Mattoccia S. Non-linear Parametric Bayesian Regression for Robust Background Subtraction.

[53] Colmenarejo A., Escudero-Violo M., Bescs J. (2011). Class-driven Bayesian background modeling for video object segmentation. Electron. Lett..

[54] Messelodi S., Modena C., Segata N., Zanin M. A Kalman Filter Based Background Updating Algorithm Robust to Sharp Illumination Changes.

[55] El Baf F., Bouwmans T., Vachon B. Type-2 fuzzy Mixture of Gaussians Model: Application to Background Modeling.

[56] Wan Zaki W.M.D., Hussain A., Hedayati M. (2011). Moving object detection using keypoints reference model. EURASIP J. Image Video Process..

[57] Anderson D., Keller J., Skubic M., Chen X., He Z. Recognizing Falls from Silhouettes.

[58] Rougier C., Meunier J., St-Arnaud A., Rousseau J. Fall Detection from Human Shape and Motion History Using Video Surveillance.

[59] Foroughi H., Aski B.S., Pourreza H. Intelligent Video Surveillance for Monitoring Fall Detection of Elderly in Home Environments.

[60] Rougier C., Meunier J., St-Arnaud A., Rousseau J. (2011). Robust video survellience for fall detection based on human shape deformation. IEEE Trans. Circuits Syst. Video Technol..

[61] Wu G. (2000). Distinguishing fall activities from normal activities by velocity characteristics. Elsevier J. Biomech..

[62] Lin C.W., Ling Z.H. Automatic Fall Incident Detection in Compressed Video for Intelligent Homecare.

[63] Liao Y.T., Huang C.L., Hsu S.H. (2012). Slip and fall event detection using Bayesian Belief Network. Pattern Recognit..

[64] Olivieri D.N., Conde I.G., Sobrino X.A. (2012). Eigenspace-based fall detection and activity recognition from motion templates and machine learning. Expert Syst. Appl..

[65] Yu M., Rhuma A., Naqvi S.M., Wang L., Chambers J. (2012). A posture recognition-based fall detection system, for monitoring and elderly person in a smart home environment. IEEE Trans. Inf. Technol. Biomed..

[66] Jansen B., Deklerck R. Context Aware Inactivity Recognition for Visual Fall Detection.

[67] Rougier C., Meunier J. (2010). 3D head trajectory using a single camera. Int. J. Future Gener. Commun. Netw..

[68] Foroughi H., Rezvanian A., Paziraee A. Robust Fall Detection using Human Shape and Multi Class Support Vector Machine.

[69] Thome N., Miguet S. A HHMM-Based Approach for Robust Fall Detection.

[70] Juang C.-F., Chang C.-M. (2007). Human body posture classification by neural fuzzy network and home care system applications. IEEE Trans. Syst. Man. Cybern. A.

[71] Liu C.-L., Lee C.-H., Lin P.M. (2010). A fall detection system using k-nearest neighbor classifier. Expert Syst. Appl..

[72] Ibrahim N., Mustafa M.M., Mokri S.S., Siong L.Y., Hussain A. (2012). Detection of snatch theft based on temporal differences in motion flow field orientation histograms. Int. J. Adv. Comput. Technol..

[73] Goya K., Zhang X., Kitayama K. A Method for Automatic Detection of Crimes for Public Security by Using Motion Analysis.

[74] Shi J., Tomasi C. Good features to track.

[75] Lucas B., Kanade T. An Iterative Image Registration Technique with an Application to Stereo Vision.

[76] Silveira Jacques J.C., Braun A., Soldera J., Musse S.R., Jung C.R. (2007). Understanding people motion in video sequences using Voronoi diagrams. Patt. Anal. Appl..

[77] Liu X., Chua C.-S. (2006). Multi-agent activity recognition using observation decomposed hidden Markov models. J. Image Vis. Comput..

[78] Wiliem A., Madasu V., Boles W., Yarlagadda P. A context-based approach for detecting suspicious behaviors.

[79] Gowun J., Yang H.S. Context Aware Activity Recognition by Markov Logic Networks of Trained Weights.

[80] Comaniciu D., Meer P. (2002). Mean shift: A robust approach toward feature space analysis. Patt. Anal. Mach. Intell..

[81] Leichter I., Lindenbaum M., Rivlin E. (2009). Mean Shift tracking with multiple reference color histograms. Comput. Vis. Image Underst..

[82] Namboodiri V.P., Ghorawat A., Chaudhuri S. (2006). Improved Kernel-Based Object Tracking Under Occluded Scenarios. Lect. Note. Comput. Sci..

[83] Comanicio D., Ramesh V., Meer P. (2003). Kernel-based object tracking. IEEE Trans. Pattern Anal. Mach. Intell..

[84] Elhamod M., Levine M.D. (2013). Automated real-time detection of potentially suspicious behavior in public transport areas. IEEE Trans. Intell. Transp. Syst..

[85] Isard M., Blake A. (1998). Condensation: Conditional density propagation for visual tracking. Int. J. Comput. Vis..

[86] Natarajan P., Nevatia R. Online, Real-time Tracking and Recognition of Human Actions.

[87] Fortmann T.E., Bar-Shalom Y., Scheffe M. (1983). Sonar tracking of multiple targets using joint probabilistic data association. IEEE J. Oceanic Eng..

[88] Pham N.T., Huang W., Ong S.H. Tracking Multiple Objects using Probability Hypothesis Density Filter and Color Measurements.

[89] Reid D.B. (1979). An algorithm for tracking multiple targets. IEEE Trans. Autom. Control.

[90] Singh S., Velastin S.A., Ragheb H. MuHAVi: A Multi-camera Human Action Video Dataset for the Evaluation of Action Recognition Methods.

[91] BEHAVE official website. http://homepages.inf.ed.ac.uk/rbf/BEHAVE/.

[92] CAVIAR Project dataset. http://groups.inf.ed.ac.uk/vision/CAVIAR/CAVIAR-DATA1/.

[93] Allen J.F. (1983). Maintaining knowledge about temporal intervals. Commun. ACM.

[94] Zhang Z., Tan T., Huang K. (2011). An extended grammar system for learning and recognizing complex visual events. IEEE Trans. Pattern Anal. Mach. Intell..

[95] Pei M., Si Z., Yao B., Zhu S.C. (2012). Learning and parsing video events with goal and intent prediction. Comput. Vis. Image Underst..

[96] Ryoo M.S., Aggarwal J.K. (2009). Semantic representation and recognition of continued and recursive human activities. Int. J. Comput. Vis..

[97] Gong S., Xiang T. Recognition of Group Activities Using Dynamic Probabilistic Networks.

[98] Elhamod M., Levine D.M. Real-Tie Semantics-Based detection of Suspicious Activities in Public Spaces.

[99] Zhang Y., Ge W., Chang M.-C., Liu X. Group Context Learning for Event Recognition.

[100] Veeraraghavan H., Schrater P., Papanikolopoulos N. Switching Kalman Filter-based Approach for Tracking and Event Detection at Traffic Intersections.

[101] Cheng H.Y., Hwang J.N. Multiple-target Tracking for Crossroad Traffic Utilizing Modified Probabilistic Data Association.

[102] Saleemi I., Hartung L., Shah M. Scene Understanding by Statistical Modeling of Motion Patterns.

[103] Cheng W.-C. (2012). PSO algorithm particle filters for improving the performance of lane detection and tracking systems in difficult roads. Sensors.

[104] Kalman R.E. (1960). A new approach to linear filtering and prediction problems. J. Basic Eng. D.

[105] Kumar P., Ranganath S., Weimin H., Sengupta K. (2005). Framework for real-time behavior interpretation from traffic video. IEEE Trans. Intell. Transp. Syst..

[106] Ng L.L., Chua H.S. Vision-based Activities Recognition by Trajectory Analysis for Parking Lot Surveillance.

[107] Li X., Porikli F.M. A hidden Markov Model Framework for Traffic Event Detection Using Video Features.

[108] Kratz L., Nishino K. Anomaly Detection in Extremely Crowded Scenes using Spatio-temporal Motion Pattern Models.

[109] Jiang F., Wu Y., Katsaggelos A.K. Abnormal Event Detection from Surveillance Video by Dynamic Hierarchical Clustering.

[110] Kamijo S., Matsushita Y., Ikeuchi K., Sakauchi M. (2000). Traffic monitoring and accident detection at intersections. IEEE Trans. Intell. Transp. Syst..

[111] Hsieh C.T., Hsu S.B., Han C.C., Fan K.-C. (2011). Abnormal event detection using trajectory features. J. Inf. Technol. Appl..

[112] Gong S., Xiang T. Recognition of Group Activities Using Dynamic Probabilistic Networks.

[113] Jimnez-Hernndez H., Gonzlez-Barbosa J.-J., Garcia-Ramrez T. (2010). Detecting abnormal vehicular dynamics at intersections based on an unsupervised learning approach and a stochastic model. Sensors.

[114] Kamijo S., Harada M., Sakauchi M. An Incident Detection System based on Semantic Hierarchy.

[115] Ivanov Y.A., Bobick A.F. (2000). Recognition of visual activities and interactions by stochastic parsing. IEEE Trans. Pattern Anal. Mach. Intell..

[116] Chen X., Zhang C. Incident Retrieval in Transportation Surveillance Videos—An Interactive Framework.

[117] Veeraraghavan H., Papanikolopoulos N.P. (2009). Learning to recognize video-based spatiotemporal events. IEEE Trans. Intell. Transp. Syst..

[118] Anderson D., Luke R.H., Keller J.M., Skubic M., Rantz M.J., Aud M.A. (2009). Modeling human activity from voxel person using fuzzy logic. IEEE Trans. Fuzzy Syst..

[119] Cucchiara R., Prati A., Vezzani R. (2007). A multi-camera vision system for fall detection and alarm generation. J. Exp. Syst..

[120] Thome N., Miguet S., Ambellouis S. (2008). A real-time, multiview fall detection system: A LHMM-based approach. IEEE Trans. Circuits Syst. Video Technol..

[121] Holte M.B., Tran C., Trivedi M.M., Moeslund T.B. (2012). Human pose estimation and activity recognition from multi-view videos: Comparative explorations of recent developments. IEEE J. Sel. Top. Signal Process..

[122] Auvinet E., Multon F., Saint-Arnaud A., Rousseau J., Meunier J. (2011). Fall detection with multiple cameras: An occlusion-ressistant method based on 3-d silhouette vertical distribution. IEEE Trans. Inf. Technol. Biomed..

[123] Shieh W.Y., Huang J.C. (2012). Falling-incident detection and throughput enhancement in a multi-camera video-surveillance system. Med. Eng. Phys..

[124] Weinland D., Ronfard R., Boyer E. (2006). Free viewpoint action recognition using motion history volumes. Comput. Vis. Image Underst..

[125] Denman S., Fookes C., Cook J., Davoren C., Mamic A., Farquharson G., Chen D., Chen B., Sidharan S. Multi-view Intelligent Vehicle Surveillance System.

[126] Weinland D., Boyer E., Ronfard R. Action Recognition fro Arbitrary Views using 3d Exemplars.

[127] Taj M., Cavallaro A. Multi-Camera Scene Analysis using an Object-Centric Continuous Distribution Hidden Markov Model.

[128] Calderara S., Cucchiara R., Prati A. A Distributed Outdoor Video Surveillance System for Detection of Abnormal People Trajectories.

[129] Adam A., Rivlin E., Shimshoni I., David R. (2008). Robust real-time unusual event detection using multiple fixed-location monitors. IEEE Trans. Pattern Anal. and Mach. Intell..

[130] Antonakaki P., Kosmopoulos D., Perantonis S.J. (2009). Detecting abnormal human behavior using multiple cameras. Signal Process..

[131] Uson P.C., Hagihara K., Ruiz D., Macq B. Towards a Visual-hull Based Multi-agent Surveillance System.

[132] Drews P., Quintas J., Dias J., Andersson M., Nygrds J., Rydell J. Crowd Behavior Analysis under Cameras Network Fusion using Probabilistic Methods.

[133] Babaei P., Fathy M. Abnormality detection and traffic flow measurement using a hybrid scheme of SIFT in distributed multi camera intersection monitoring.

[134] Hoai M., De la Torre F. Max-margin early event detectors.

[135] Ryoo M.S. Human Activity Prediction: Early Recognition of Ongoing Activities from Streaming Videos.

[136] Li W., Zhang W., Liu Z. (2008). Expandable data-driven graphical modeling of human actions based on salient postures. IEEE Trans. Circuits Syst. Video Technol..

